# Rhenium Perrhenate (^188^ReO_4_) Induced Apoptosis and Reduced Cancerous Phenotype in Liver Cancer Cells

**DOI:** 10.3390/cells11020305

**Published:** 2022-01-17

**Authors:** Samieh Asadian, Abbas Piryaei, Nematollah Gheibi, Bagher Aziz Kalantari, Mohamad Reza Davarpanah, Mehdi Azad, Valentina Kapustina, Mehdi Alikhani, Sahar Moghbeli Nejad, Hani Keshavarz Alikhani, Morteza Mohamadi, Anastasia Shpichka, Peter Timashev, Moustapha Hassan, Massoud Vosough

**Affiliations:** 1 Cellular and Molecular Research Center, Research Institute for Prevention of Non-Communicable Diseases, Qazvin University of Medical Sciences, Qazvin 34199153, Iran; samieh.asadian@gmail.com (S.A.); haematologicca@gmail.com (M.A.); smoghbelinrjad@qums.ac.ir (S.M.N.); 2 Department of Regenerative Medicine, Cell Science Research Center, Royan Institute for Stem Cell Biology and Technology, ACECR, Tehran 16635148, Iran; alikhanim81@gmail.com (M.A.); hani.keshavarz865@gmail.com (H.K.A.); 3 Department of Biology and Anatomical Sciences, School of Medicine, Shahid Beheshti University of Medical Sciences, Tehran 16123798, Iran; piryae_a@yahoo.com; 4 Department of Tissue Engineering and Applied Cell Sciences, School of Advanced Technologies in Medicine, Shahid Beheshti University of Medical Sciences, Tehran 16123798, Iran; 5 Department of Organic Chemistry, Karaj Branch, Islamic Azad University, Karaj 16255879, Iran; b_akalantari@yahoo.com; 6 Faculty of Nuclear Engineering, Shahid Beheshti University, Tehran 15689456, Iran; behzad_davar2001@yahoo.com; 7 Department of Internal Medicine N1, Sechenov University, 119991 Moscow, Russia; kapustina_v_a@staff.sechenov.ru; 8 Department of Physical Chemistry, Faculty of Science, University of Tehran, Tehran 17456987, Iran; sarvar20102010@gmail.com; 9 World-Class Research Center “Digital Biodesign and Personalized Healthcare”, Sechenov University, 119991 Moscow, Russia; ana-shpichka@yandex.ru; 10 Institute for Regenerative Medicine, Sechenov University, 119991 Moscow, Russia; 11 Chemistry Department, Lomonosov Moscow State University, 119991 Moscow, Russia; 12 Experimental Cancer Medicine, Institution for Laboratory Medicine, Karolinska Institute, 141-83 Stockholm, Sweden; Moustapha.Hassan@ki.se; 13 Clinical Research Center, Karolinska University Hospital Huddinge, 141-83 Stockholm, Sweden

**Keywords:** hepatocellular carcinoma, apoptosis induction, cell cycle arrest, Rhenium-188 (^188^Re), radionuclide therapy

## Abstract

Recurrence in hepatocellular carcinoma (HCC) after conventional treatments is a crucial challenge. Despite the promising progress in advanced targeted therapies, HCC is the fourth leading cause of cancer death worldwide. Radionuclide therapy can potentially be a practical targeted approach to address this concern. Rhenium-188 (^188^Re) is a β-emitting radionuclide used in the clinic to induce apoptosis and inhibit cell proliferation. Although adherent cell cultures are efficient and reliable, appropriate cell-cell and cell-extracellular matrix (ECM) contact is still lacking. Thus, we herein aimed to assess ^188^Re as a potential therapeutic component for HCC in 2D and 3D models. The death rate in treated Huh7 and HepG2 lines was significantly higher than in untreated control groups using viability assay. After treatment with ^188^ReO_4_, Annexin/PI data indicated considerable apoptosis induction in HepG2 cells after 48 h but not Huh7 cells. Quantitative RT-PCR and western blotting data also showed increased apoptosis in response to ^188^ReO_4_ treatment. In Huh7 cells, exposure to an effective dose of ^188^ReO_4_ led to cell cycle arrest in the G2 phase. Moreover, colony formation assay confirmed post-exposure growth suppression in Huh7 and HepG2 cells. Then, the immunostaining displayed proliferation inhibition in the ^188^ReO_4_-treated cells on 3D scaffolds of liver ECM. The PI3-AKT signaling pathway was activated in 3D culture but not in 2D culture. In nude mice, Huh7 cells treated with an effective dose of ^188^ReO_4_ lost their tumor formation ability compared to the control group. These findings suggest that ^188^ReO_4_ can be a potential new therapeutic agent against HCC through induction of apoptosis and cell cycle arrest and inhibition of tumor formation. This approach can be effectively combined with antibodies and peptides for more selective and personalized therapy.

## 1. Introduction

Hepatocellular carcinoma (HCC), the dominant variety of liver cancers, represents the sixth prevalent and fourth leading cause of cancer death worldwide [1]. The main risk factors for HCC are chronic infection with hepatitis C virus (HCV) or hepatitis B virus (HBV), consumption of aflatoxin-contaminated foods, alcohol abuse, obesity, smoking, and type 2 diabetes [1]. HCC staging is critical for predicting survival and selecting the best treatment strategy. Therapeutic strategies, such as hepatectomy, hepatic artery ligation and catheterization, transarterial chemoembolization (TACE), using multi-kinase inhibitors (Sorafenib), and external radiation therapy, may all result in tumor down-staging [2]. For less advanced HCC, potentially curative therapies, such as liver transplantation or surgical resection of the tumor, might be an appropriate approach [3]. Following resection, metastasis and recurrence are common, reducing overall survival. In patients with unresectable HCC and preserved liver function, TACE can prolong the survival rate. However, TACE is rarely curative [4]. Due to the partially beneficial nature of current chemotherapy or alternative medicine, the development of novel treatments for liver cancer, especially advanced HCC, is of urgent need [4]. 

Selective radionuclide therapy, which contains yttrium-90 microspheres, iodine-131 monoclonal antibody, and radioactive lipiodol, is an effective local treatment for HCC [5]. Radioisotopes that continuously emit β-rays can destruct tumor cells by frequent low-dose radiation after intra-tumoral injection [2]. Rhenium-188 (^188^Re) is an easily-available generator-derived radioisotope for therapeutic application that emits β-particles (2.12 MeV, 71.1% and 1.965 MeV, 25.6%) and image-able gammas (155 keV, 15.1%) [5]. ^188^Re has been used to prepare therapeutic radiopharmaceuticals for the management of different diseases, such as bone metastasis, rheumatoid arthritis, and primary cancers [5]. 

The activity of anticancer drugs has been evaluated in cancer cell lines cultured under two-dimensional (2D) condition. Although 2D cell cultures have provided valuable insight into tumor cell proliferation, they do not reveal the complex interactions that exist between the cancer cells and their microenvironment [6]. This could lead to a misinterpretation of experimental findings collected in a 2D condition culture. The application of 3D culture systems as a proper platform can avoid certain drawbacks by recapitulating the tumor microenvironment. It has been shown that 3D organotypic human cancer cell models, as well as hepatocyte 3D constructs, are suitable alternatives to adherent cultures in terms of producing more valid data [7]. They can be tailored to be biomimetic in order to accurately recapitulate the native in vivo environment. Accordingly, rat hepatocytes in 3D cultures present structural polarity and possess channels with great similarity in structure and function to bile canaliculi, which can explain their enhanced hepatocellular activity [7]. In contrast to normal cells, tumor cells with stem cell features, such as EpCAM + human HCC cells, can also generate 3D spheroids [8].

A large panel of HCC cell lines with different mutational profiles would cover a great portion of the genomic heterogeneity of primary HCCs. Careful selection would determine whether these cell lines mimic responses of primary HCCs to different drugs and may lead to the development of a powerful cell line-based platform for the successful treatment of HCCs. Here, we investigated ^188^ReO_4_ inhibitory effects on cancer development in Huh7 and HepG2 malignant HCC cell lines.

Single and double DNA break stimulation results in activation of apoptosis or cell cycle arrest as a part of negative feedback response to limit proliferation [9]. Thus, β−particle irradiation in 2D, 3D, and mouse models results in DNA damage [10,11,12]. Enhancing apoptosis signaling in-vitro and in-vivo appears to be a consequence of both increased single- and double-strand DNA break caused by therapeutic radionuclide accumulation. This has a positive effect on the elimination of malignant cells [13]. Activation of p53 and BAX following exposure to β−emission is mediated by DNA damage and apoptosis induction [5]. However, the effects of ^188^ ReO_4_ on apoptosis, cell cycle arrest, and in vitro and in vivo growth of cancerous HCC cells have not been well studied.

The 3D cell culture systems are based on matrices made from natural materials, such as alginate, collagen, and glycosaminoglycan, as well as synthetic sources, such as polyethylene glycol and polyacrylic acid [14,15]. Recently, decellularized extra-cellular matrix (ECM)-derived materials have been extensively used in tissue engineering and regenerative medicine. These materials usually preserve the original composition and partial structure of tissue-specific ECM [16]. 

In recent times, researchers have successfully used decellularized ECM materials to develop 3D cancer models [17]. Therefore, culturing HCC cells on the decellularized liver extra-cellular matrix (LEM) might provide a better biomimetic microenvironment for them. Moreover, HepG2 is commonly employed as a surrogate tool of primary hepatocytes for toxicological and pharmacological research. HepG2 is specifically useful for studying toxicities of chemicals that affect DNA replication and cell cycling since it can take several cell passages before the threshold of toxic effect is reached [18]. Huh7 has also been used in toxicity and drug metabolism research [19,20]. In this study, we cultured Huh7 and HepG2 hepatic cell lines on 3D scaffolds derived from LEM hydrogel and called them Huh7/HepG2-LEM. To investigate the effect of ^188^ReO_4_, we focused on PTEN/PI3K/AKT signaling pathway to assess whether ^188^ReO_4_ affects radioresistance in vitro. 

## 2. Materials and Methods

### 2.1. Cell Culture

HCC cell lines, including Huh7 and HepG2, were obtained from Royan Cell Bank. We used these two different cell lines as HCC models to consider the various metabolic profile, cancerous phenotype, and functional assessments, as well as individual diversity, in response to HCC treatment. The cells were cultured in high-glucose Dulbecco’s modified Eagle’s medium (HG-DMEM, Gibco, 11995-040, New York, NY, USA) at 37 °C in a humidified incubator with 5% CO_2_. The culture medium was supplemented with 10% fetal bovine serum (FBS, Gibco, 16140-071), 2 mM L-glutamine (Gibco, 25030-024), 1 mM non-essential amino acids (Gibco, 11140-035), and 1% penicillin/streptomycin (Pen/Strep, Gibco; 15070-063, Gaithersburg, MD, USA). Huh7 and HepG2 cells were sub-cultured by trypsin/EDTA (0.25%, Gibco; 25,200,056). The culture media were changed every other day.

### 2.2. Generation of HCC-LEM

Since necessary cell-cell and cell-ECM interactions are preserved, cancer cells can better maintain their phenotype in 3D condition. Sheep LEM was already produced in our lab [21]. Briefly, sheep liver was frozen, chopped into small pieces, and subjected to mechanical agitation for 2.5 h in distilled water. The slices were then stirred in 1% sodium dodecyl sulfate (SDS, Sigma, L3771, St. Louis, MO, USA) at 4 °C for 36 h. Finally, decellularized pieces were stirred in deionized water at 4 °C for 12 h, to remove the detergents and cellular fragments. To generate HCC-LEM, 1.5 × 10^5^ Huh7 or HepG2 cells were seeded on the scaffold derived from LEM-hydrogel. Then, both 3D constructs were cultured for 10 days.

### 2.3. Cell Proliferation Assay

To find the half-maximal inhibitory concentration (IC50), a total of 2 × 10^4^ cells/well were seeded in 96-well plates. At 75–80% confluency, Huh7 cells were treated with various doses of 18, 37, and 55 MBq of ^188^ReO_4_, whereas HepG2 cells were treated with 37, 55, and 73 MBq of ^188^ReO_4_ (PARS-ISOTOPE Company, Tehran, Iran). After administration of ^188^ReO_4_ at different time-points (18, 24, and 48 h), 10 µL of ORANGU solution (Cell Guidance Systems; OR01-500, Cambridge, UK) was added to each well. The plate was incubated at 37 °C for 2 h. The absorbance values were measured at 450 nm using a Stat Fax microplate reader (Awareness Technology, Inc., Palm City, FL, USA). According to the kit datasheet, cell proliferation is proportional to an optical density (OD).

### 2.4. Viability Assay

Cells were cultured in a 2D condition and exposed to the IC50 dose of ^188^ ReO_4_ at three time points (18, 24, and 48 h), harvested, and suspended in PBS. Finally, cells were evaluated by LIVE/DEAD^®^ Viability/Cytotoxicity Kit (Invitrogen, L3224, Waltham, MA, USA). The nuclei were counterstained with DAPI and viability was determined using a fluorescence microscope (Olympus IX71, Tokyo, Japan).

### 2.5. Cell Cycle Analysis

Equal number of cells were dissociated using trypsin/EDTA (0.25%, Gibco; 25,200,056). Cells were fixed in 500 μL of 70% EtOH overnight at −20 °C. Fixed cells were washed and resuspended in FxCycle PI/RNase Staining Solution (ThermoFisher, Waltham, MA, USA) at a concentration of 10^6^ cells/mL, and then incubated at room temperature (RT) for 20 min in a dark place before flow cytometry analysis (FACSCalibur, BD, Franklin Lakes, NJ, USA). The data were analyzed using FlowJo 10.6.2.

### 2.6. Apoptosis Assessment by Flow Cytometric Analysis

In the 2D culture system, the apoptosis rate was determined in control and treatment groups using flow cytometric analysis. Three replicates of 1 × 10^5^ Huh7 or HepG2 cells were incubated with fluorescein isothiocyanate (FITC)-conjugated Annexin V and propidium iodide (556,547, Annexin V-FITC apoptosis detection kit, BD Pharmingen; BD Biosciences, Franklin Lakes, NJ, USA) on ice for 30 min, 18, 24, and 48 h after exposure to the IC50 dose of ^188^ReO_4_. Then, the cells were washed three times with PBS and 400 µL binding buffer was added to the cells. The cell suspension was analyzed by FACSCalibur. The data were analyzed using BD CellQuest™ Pro software (version 5.2.1; BD Biosciences), and the percentage of apoptotic cells per group was calculated.

### 2.7. Quantitative Reverse Transcriptase–Polymerase Chain Reaction (qRT-PCR)

To evaluate apoptosis and the radio-resistance signaling pathway in Huh7 /HepG2 cells treated with IC50s at the transcriptional level, we performed qRT-PCR for *p53*, *BAX*, *PTEN*, and *PI3K* genes. RNA extraction was performed using TRIzol (Invitrogen^®^), and cDNA was synthesized using PrimeScript^™^ Reverse Transcriptase Kit (Takara Bio, Inc., Kusatsu-Shi, Japan) according to the instructions provided by the manufacturer. Quantitative RT-PCR reactions were performed by a real-time PCR system (Applied Biosystems StepOne instrument, Foster City, CA, USA) using SYBR Green Master Mix (SYBR Premix, Takara Bio, Inc., Kusatsu, Japan), and the results were analyzed by StepOne software (Applied Biosystems; version 2.1). For each group, the samples were collected from three independent biological replicates. Finally, the expression levels of each target genes were normalized against *GAPDH.* Analysis was performed by the comparative CT Method 2^−ΔΔ3t^.

### 2.8. Histological Assessment and Immunofluorescence Assay

Samples were harvested from both 3D structures at the selected time points post-exposure, fixed in 4% formaldehyde overnight, and embedded in paraffin blocks. Then, 5-µm sections were prepared. To scrutinize the microstructure of the samples, hematoxylin and eosin (H&E) staining was performed. In addition, to evaluate the expression of apoptosis-related proteins, radio-resistance, and proliferation-related proteins in both 3D constructs, immunostaining was performed to detect p53, BAX, P-AKT, and Ki67. The sections were incubated overnight at 4 °C with primary antibodies, including mouse monoclonal antibody p53 (Santa Cruz, sc-126, Dallas, TX 75220, USA), BAX (Santa Cruz, sc-7480), rabbit polyclonal antibody P-AKT (Santa Cruz, sc-135650), and rabbit monoclonal antibody Ki67 (Cell Signaling Technology, 9129, Danvers, MA, USA). Then, the sections were incubated with secondary antibody for 1 h at 37 °C. The nuclei were counterstained with DAPI, and the slides were analyzed under a fluorescent microscope (Olympus BX51).

### 2.9. Immunoblotting Analysis

The 2D-treated and control cells were lysed in 1 × RIPA buffer (Sigma-Aldrich, St. Louis, MS, USA) supplemented with Halt^™^ protease and phosphatase inhibitor cocktail (ThermoFisher Scientific, Waltham, MA, ISA). Protein concentration was determined by the Bradford method. Equal concentrations of protein extract were subjected to 12% SDS-PAGE and transferred to poly vinylidene fluoride (PVDF) membrane. Blots were labeled with the primary antibodies, p53 (1:500, Santa Cruz, sc-126), BAX (1:500, Santa Cruz, sc-7480), and P-AKT (1:500, Santa Cruz, sc-135650). β-actin was used as the control. Then, the membranes were washed with 0.1% Tween-20 TBS (pH 7.6) and incubated with horseradish peroxidase (HRP)-conjugated secondary antibody (1:5000, Sigma-Aldrich), and blots were incubated for 3 h at RT. Immune-reactive protein bands were detected using enhanced chemiluminescence (ECL) reagent (Bio-Rad, Hercules, CA, USA), and membranes were imaged by ImageQuant LAS 4000 mini, GE Healthcare. The densitometry of each band was measured by ImageJ. The densitometry results represent the mean ± SEM.

### 2.10. Hepatocellular Carcinoma (HCC) Mouse Model

To evaluate tumorigenicity of the available cells, Huh7 cells at different numbers (3 × 10^6^, 5 × 10^6^, 7 × 10^6^, and 10 × 10^6^) were subcutaneously injected to four anatomical sites (flanks and shoulders) in nude mice. When the tumors reached a visible size, the recipient nude mouse scarified following the ethical guidelines, and tumors were removed, and their dimensions were measured (Supplementary Figure S1). All of the experiments with animals were performed under the Guide for the Care and Use of Laboratory Animals (National Institutes of Health Publication No. 80-23, revised 1996) and approved by the Research and Ethics Committee of Royan Institute. (approval No. IR.ACECR.ROYAN.REC.1397.052).

### 2.11. Tumor Formation Assay

To assess whether ^188^ReO_4_-treated cells could form any tumor, Huh7 cells were exposed to 37 MBq for 24 h. A total number of 5 × 10^6^ cells were percutaneously injected to the flank of nude mice. As a control group, the same number (5 × 10^6^) of untreated Huh7 cells was percutaneously injected to the flank of other nude mice (*n* = 3). After 14 days, animals were sacrificed for further assessments (Supplementary Figure S1A).

### 2.12. Statistical Analysis

All statistical analyses were performed using GraphPad-Prism (version 6). Data are presented as mean ± standard deviation (SD). The Kolmogorov-Smirnov normality tests were performed to examine the normal distribution of the data. Differences among groups were assessed using one-way repeated measures analysis of variance (ANOVA), and the LSD method done for post hoc multiple comparisons. *p* < 0.05 was considered a statistically significant difference.

## 3. Results

### 3.1. Assessment of 2D Construction

#### 3.1.1. ^188^ReO_4_ Treatment Reduced Viability in 2D Cultured Liver Cancer Cells

**Figure 1:**
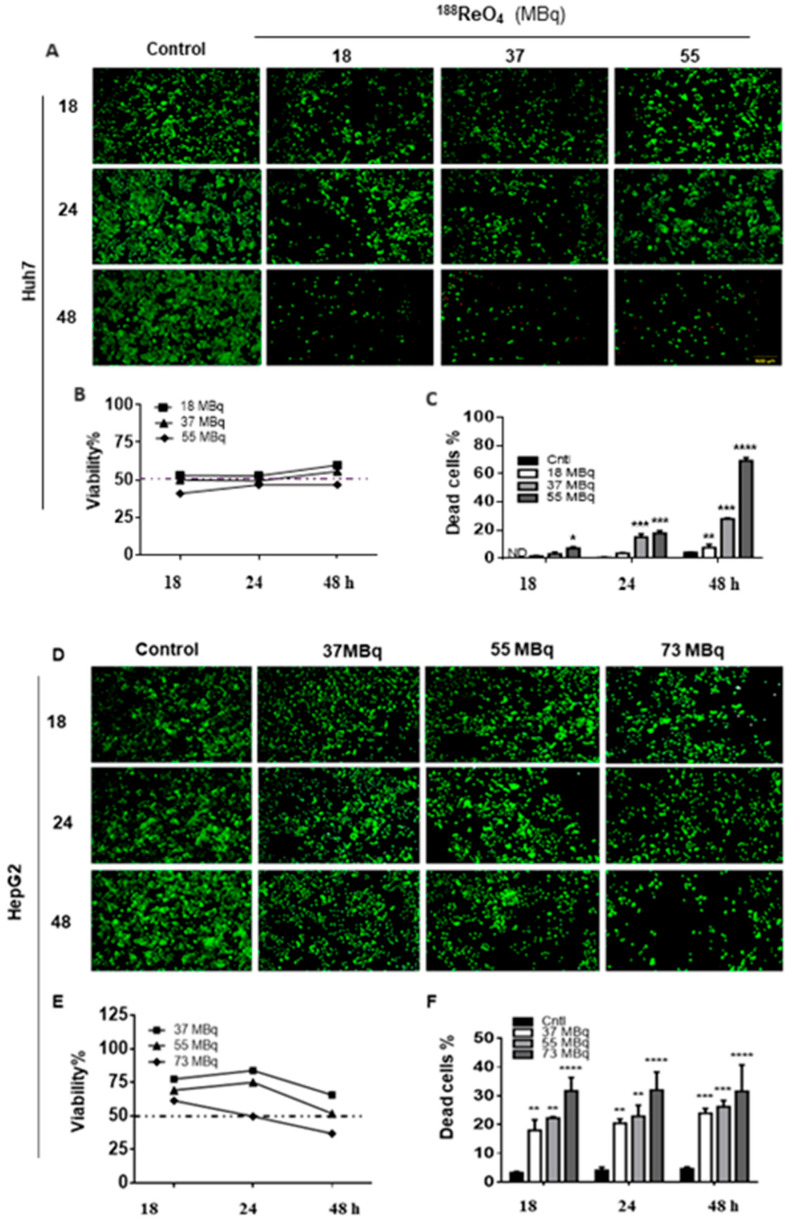
^188^ReO_4_ IC50 dose finding on Huh7 and HepG2 cell lines. Huh7 cells viability was measured using LIVE/DEAD^®^ Viability/Cytotoxicity Kit and the mean viability of untreated cells (control group), and the treated groups were compared on various doses of 18, 37, and 55 MBq of ^188^ReO_4_ at 18, 24, and 48 h post-exposure for finding the effective dose of ^188^ReO_4_ (**A**–**C**). HepG2 cells viability was measured using LIVE/DEAD^®^ Viability/Cytotoxicity Kit in response to 37, 55, and 73 MBq of ^188^ReO_4_ 18, 24, and 48 h post-exposure for finding the effective dose of ^188^ReO4 in treated HepG2 cells (**D**–**F**). The IC50 value of 188ReO_4_ in Huh7 cells was 37 MBq 24 h after exposure, and it was 55 MBq 48 h for HepG2 cells. Data are presented as the mean ± SD, *n* = 3 (* *p* < 0.05, ** *p* < 0.01, and *** *p* < 0.001, **** *p* < 0.0001).

We performed a live/dead cell assay to determine changes in cell viability after ^188^ReO_4_ administration. As shown in Figure 1A,D, the ^188^ReO_4_ radionuclide exposure changed cell proliferation rate and viability. There was a difference in dead cell percentage among different radionuclide activities and among different time points in the two cell lines. The highest percentage of dead cells was 69.19% following treatment with 55MBq of ^188^ReO_4_ (*p* < 0.0001) and 44.1% following administration of 73MBq of ^188^ReO_4_ (*p* < 0.0001), 48 h after exposure for Huh7 and HepG2 cells, respectively. In our previous published paper, the impact of ^188^ReO_4_ exposure on HDF as a normal cell has been demonstrated. We reported that the viability of HDF cells did not change after incubation with ^188^ReO_4_ [22]. As shown in Figure 1B,E, ^188^ReO_4_ cytotoxicity in human liver cancer cell lines was dose-dependent. Notably, both hepatoma cell lines exhibited different sensitivities to ^188^ReO_4_. While the IC50 value of ^188^ReO_4_ in Huh7 cells was 37 MBq 24 h after exposure, it was 55 MBq 48 h for HepG2 cells. Finally, 37 and 55 MBq were chosen as IC50 dose for Huh7 and HepG2 cells, respectively, to perform further analysis.

#### 3.1.2. ^188^ReO_4_ Induced G2/M Arrest in Huh7 Cancer Cells but Not HepG2

**Figure 2:**
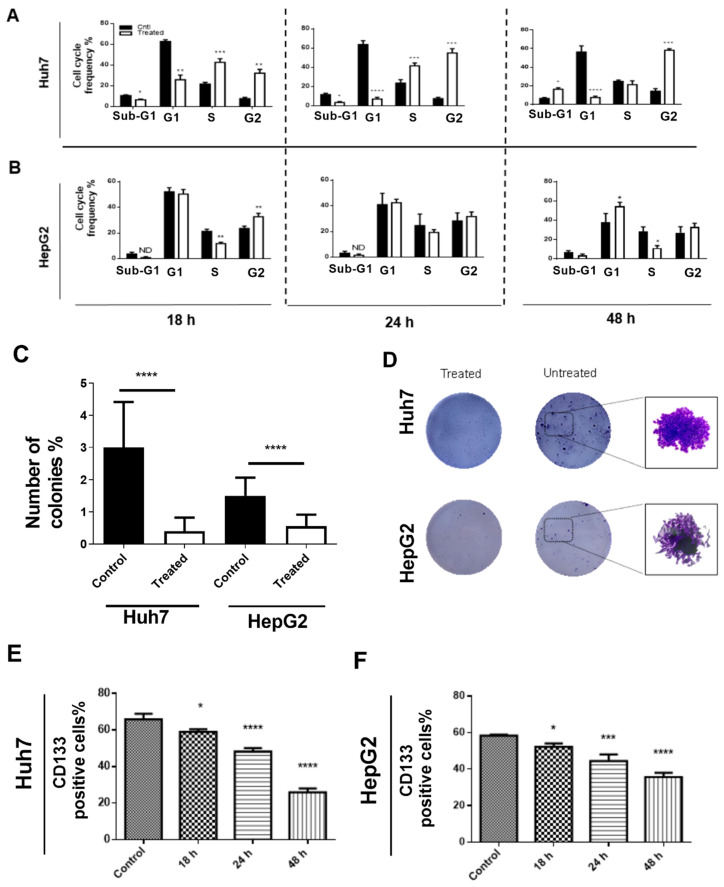
Cell cycle evaluation and colony formation assay. (**A**,**B**) Flow cytometry evaluated cell cycle profile for cells treated with 37 MBq (Huh7)/55 MBq (HepG2) of ^188^ReO_4_ versus untreated group 18, 24, and 24 h post-exposure using PI/ RNase staining. The results showed that 37 MBq exposure led into significant G2/M arrest in Huh7 cells after 24 h, while, in HepG2, 55MBq exposure did not make significant changes in cell cycle phases. (**C**,**D**) Cells treated with 37 MBq (Huh7)/55 MBq (HepG2) of ^188^ReO_4_ reduced colony formation capacity. The results show an almost three-fold lower number of colonies in treated cells compared to untreated control cells. (**E**,**F**) The number of CD133 positive cells decreased significantly after treatment in a time dependent manner. Data are expressed as the mean ± SD, *n* = 3 (* *p* < 0.05, ** *p* < 0.01, and *** *p* < 0.001, **** *p* < 0.0001) versus the control group.

The effect of ^188^ReO_4_ at IC50 doses on the cell cycle progression was assessed in both cell lines after 18, 24, and 48 h of exposure. FACS analysis showed that 37 MBq exposure resulted in significant G2/M arrest in Huh7 cells after 24 h (*p* < 0.001), while, in HepG2, 55MBq exposure did not make significant changes in cell cycle phases (*p* > 0.05) (Figure 2A,B). However, after 48 h of exposure, Huh7 cells either entered sub-G1 phase (cell death) or reverted to normal cell cycle. Interestingly, G2/M arrest was not observed in HepG2 cells. Cell cycle analysis after exposure to 55 MBq in HepG2 cells showed reduction of cells number in S phase (*p* < 0.01) with a concomitant increase in the number of cells in G2 (*p* < 0.01) and G1 phase of the cell cycle, with minor impact on the sub-G1 phase at 18 and 48 h post-exposure. 

Furthermore, to evaluate the effects of ^188^ReO_4_ treatment on colony formation capability, Huh7 and HepG2 cells, respectively, treated with 37 and 55 MBq. Data showed an almost three-fold lower number of colonies than untreated control cells in the plates (*p* < 0.001; Figure 2C,D).

#### 3.1.3. ^188^ReO_4_ Induced Apoptosis in Hepatic Cancer Cells

**Figure 3:**
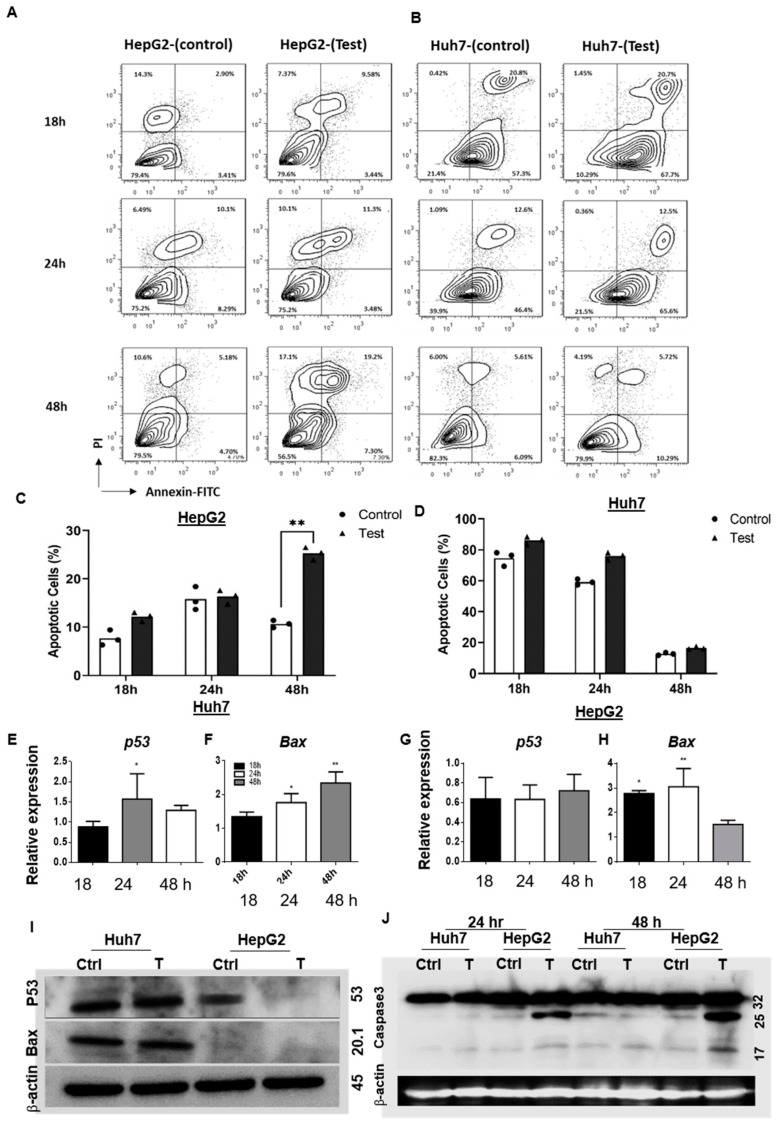
Apoptosis induction evaluation. (**A**,**B**) Apoptosis evaluated using Annexin PI at three time points of 18, 24, and 48 h post-exposure for both Huh7 and HepG2 cell lines. (**C**,**D**). Bar plots show increased percentage of apoptotic Huh7 cells and HepG2 cells at 18, 24, and 48 h post-exposure. (**E**–**H**) represents the qPCR results for *p53* and *Bax* relative mRNA expression in Huh7 and HepG2 cells, respectively, treated with 37 and 55 MBq of ^188^ReO_4_ and normalized to control cells at 18, 24, and 48 h post-exposure. The *GAPDH* used as a housekeeping gene. Huh7 cells displayed increased relative expression of *p53* mRNA at 24 h and *Bax* mRNA was significantly higher in Huh7 cells at 24 and 48 h post-exposure. HepG2 cells showed increased Bax expression 18 and 24 h post-exposure. (**I**) Western blots of p53 and BAX protein in Huh7 and HepG2 cells before and 48 h after treatment. (**J**) Western blots of Caspase 3 protein in Huh7 and HepG2 cells before and 24 and 48 h after treatment. Data are expressed as the mean ± SD, *n* = 3 (* *p* < 0.05, ** *p* < 0.01).

The ^188^ReO_4_-induced apoptosis was measured using Annexin V-APC/7-AAD at 18, 24, and 48 h following exposure (Figure 3A–D). Statistically significant increase in the number of apoptotic HepG2 cells were observed 48 h post-exposure (*p* < 0.01; Figure 3A,C). Although the number of apoptotic Huh7 cells increased 18, 24, and 48 h post-exposure, it does not show any significant difference (*p* > 0.05; Figure 3A,D).

The mRNA expression of *p53* and *Bax* was analyzed after treatment with ^188^ReO_4_ in Huh7 and HepG2 cells at three time points. Compared to the control group, Huh7 cells displayed increased relative expression of *p53* mRNA 24 h post-exposure (*p* < 0.05), while, in HepG2 cells, there was no significant difference in mRNA expression at three time points following ^188^ReO_4_ exposure (Figure 3E,G). Moreover, the qRT-PCR analysis revealed that mRNA level of *Bax* was significantly higher in Huh7 cells 24 and 48 h post-exposure to 37 MBq of ^188^ReO_4_ (*p* < 0.05 and *p* < 0.01, respectively; Figure 3F). *Bax* expression 18 and 24 h post-exposure to 55 MBq of ^188^ReO_4_ significantly increased in HepG2 cells (*p* < 0.05 and *p* < 0.01, respectively; Figure 3H).

Western blot assay data showed remarkable increase in p53 and BAX protein expressions 24 h post-exposure in Huh7 cells but no remarkable changes in HepG2 cells (Figure 3I). We also assessed Caspase 3 protein development at 24 and 48 h post- exposure. The western blot results showed remarkable increase in caspase 3 protein 24 and 48 h post-exposure in HepG2 cells but not in Huh7 cells (Figure 3J).

### 3.2. Assessment of 3D Constructs

#### 3.2.1. ^188^ReO_4_ Exposure Reduced Viability in Both Cell Lines in 3D LEM Constructs 

**Figure 4:**
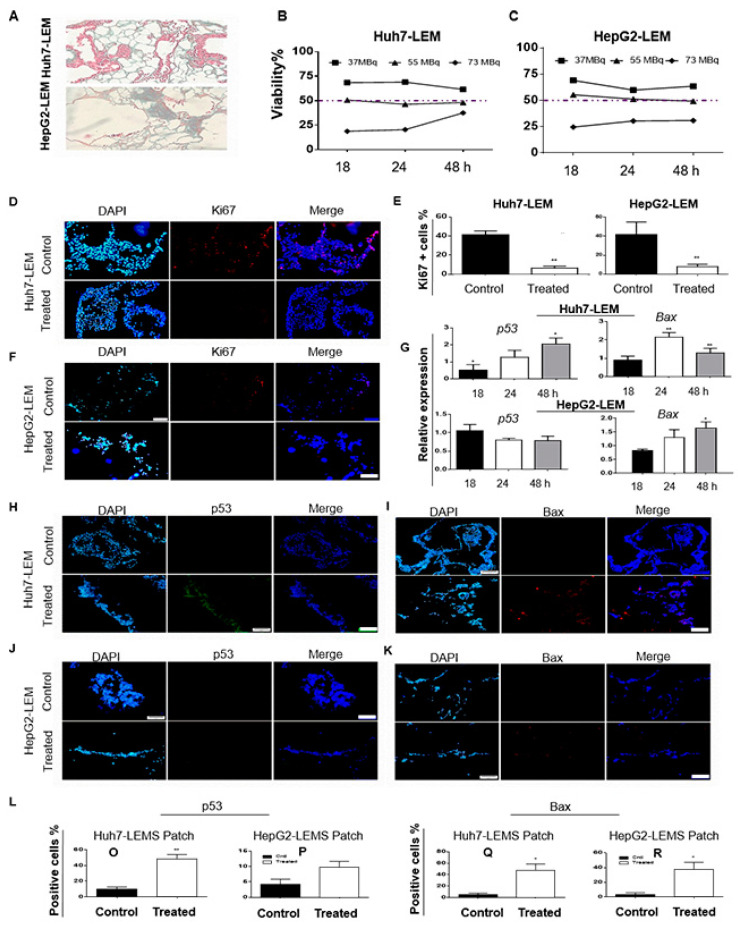
Evaluation of Huh7 and HepG2 cells in 3D LEM culture. (**A**) MT staining of Huh7 and HepG2 cells homing at synthesized LEM. (**B**,**C**) Viability percentage versus the control for finding the effective dose of ^188^ReO_4_ in Huh7/HepG2-LEM treated with 37, 55, and 73 MBq doses 18, 24, and 48 h post-exposure. (**D**–**F)** The Ki67-positive cells representing proliferating fraction of Huh7/HepG2-LEMs treated with ^188^ReO_4_. (**G**) The qPCR results for *p53* and *Bax* relative mRNA expression in Huh7/HepG2-LEMs treated with ^188^ReO_4_ versus the control after 18, 24, and 48 h. (**H**–**L**) IF staining done to visualize p53 and Bax protein expressions in Huh7-LEM and HepG2-LEM treated with ^188^ReO_4_. Scale bars: 200 μm. Values are presented as mean ± SD, *n* = 3 (* *p* < 0.05, ** *p* < 0.01).

Orangu^®^ assay performed on the Huh7-LEM and HepG2-LEM after ^188^ReO_4_ treatment at 37, 55, and 73 MBq, to determine total cell viability and effective activity dose. Samples were collected 18, 24, and 48 h following the exposure. Cell viability in both 3D constructs after exposure to 55 MBq of the radionuclide declined up to 50% (IC50) from 18 to 48 h (Figure 4B,C).

Ki-67-specific staining is visualized in Figure 4D,F. Following 48 h exposure to 55 MBq, Ki-67-positive cells in both 3D constructs decreased (*p* < 0.01; Figure 4E).

#### 3.2.2. ^188^ReO_4_ Induced Apoptosis in Cells Cultured on 3D LEM Constructs

Radionuclide exposure affected *p53* and *Bax* mRNA expression in both 3D constructs (Figure 4G). Following 18 and 48 h of exposure to 55 MBq of the radionuclide, *p53* mRNA levels in Huh7-LEM were significantly different from those of the untreated control group (*p* < 0.05). However, there were no significant differences in *p53* mRNA expression in HepG2-LEM group after exposure. To show whether exposure to 55 MBq of ^188^ReO_4_ induces apoptosis in both 3D constructs, the mRNA abundance of the pro-apoptotic gene *Bax* was quantified. Results showed significant differences in *Bax* mRNA expression in Huh7-LEM 24 and 48 h after exposure (*p* < 0.01). However, in HepG2-LEM, a significant increase in *Bax* mRNA expression was found only 48 mRNA after exposure (*p* < 0.05; Figure 4G).

To assess whether ^188^ReO_4_ exposure promotes p53 and Bax protein expression in both 3D constructs treated with 55 MBq of the radionuclide, immunofluorescence staining was performed. ^188^ReO_4_ treatment resulted in upregulation of p53 protein in Huh7-LEM compared to the control group (*p* < 0.01; Figure 4H,L), whereas there were no significant changes in p53 in HepG2-LEM (Figure 4J,L). There were increase in Bax protein post-exposure in both 3D constructs compared to their control groups (*p* < 0.05; Figure 4L). These findings suggest that ^188^ReO_4_ induced pro-apoptotic function of Bax protein in 3D constructs.

#### 3.2.3. ^188^ReO_4_ Overcame Radio-Resistance in 2D but Not in 3D Condition

**Figure 5:**
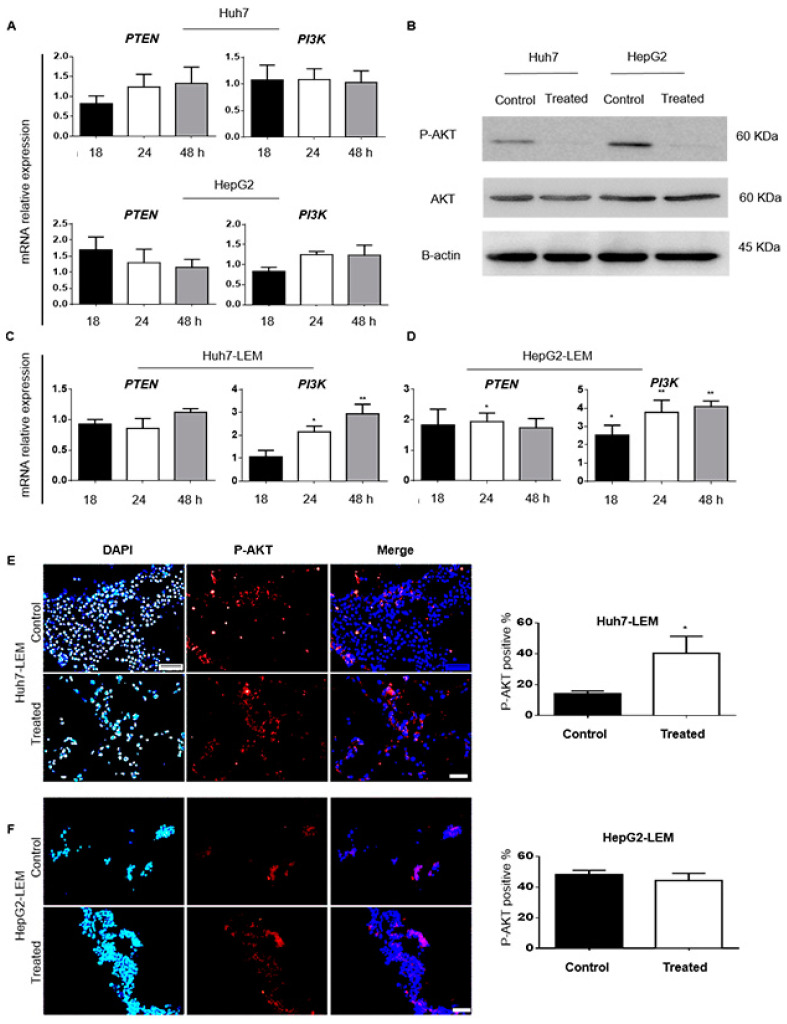
Radio-resistance evaluation. (**A**,**C**,**D**) The qPCR results for *PTEN* and *PI3K* relative mRNA expressions in 2D cultured Huh7 and HepG2 cells, respectively, treated with 37 and 55 MBq of ^188^ReO_4_, versus the control 18, 24, and 48 h post-exposure. (**B**) Western blots and relative bar graphs of P-AKT protein alternation in Huh7 and HepG2 cells, respectively, after exposure versus the untreated control group. (**E**,**F**) The qPCR results for *PTEN* and *PI3K* relative mRNA expression in Huh7/HepG2-LEMs 3D culture 18, 24, and 48 h post-exposure. Values are expressed as mean ± SD, *n* = 3 (* *p* < 0.05, ** *p* < 0.01). IF staining done to detect P-AKT expression in Huh7-LEM and HepG2-LEM treated with 55 MBq of ^188^ReO_4_ (Scale bars: 200 μm).

The qRT-PCR was performed to determine whether PTEN and PI3K contribute to radio-resistance after exposure to ^188^ReO_4_ in 2D and 3D constructs. We analyzed mRNA expression in Huh7 and HepG2 cells in 2D samples. We observed that *PTEN* and *PI3K* transcript levels were consistent after 18, 24, and 48 h exposure to 37 MBq in Huh7 and 55 MBq in HepG2 cells (Figure 5A). Then, western blot was used to determine the effects of exposure to the IC50 dose on AKT and p-AKT in 2D condition. Our data revealed that ^188^ReO_4_ exposure reduced P-AKT protein level compared to the control groups in both Huh7 and HepG2 cell lines, while it does not have any considerable impact on the total AKT level (Figure 5B).

To quantify the mRNA expression of *PTEN* and *PI3K* in 3D constructs, we first analyzed the expression levels of mRNAs following the exposure to 55 MBq of ^188^ReO_4_ at 18, 24, and 48 h. The qRT-PCR data for *PTEN* mRNA expression did not show any significant difference for 3D culture of Huh7-LEM. However, a significant increase in *PTEN* gene expression was observed in HepG2-LEM 24 h post-exposure (*p* < 0.05). The *PI3K* up-regulation was observed in Huh7-LEM and HepG2-LEM 3D constructs 24 h (*p* < 0.05 and *p* < 0.01, respectively) and 48 h (*p* < 0.01) post-exposure (Figure 5C,D).

Furthermore, P-AKT protein level was evaluated in both 3D cultured constructs following ^188^ReO_4_ exposure using IF staining. The results revealed that P-AKT protein was up-regulated after exposure to the effective dose of the radionuclide in Huh7-LEM construct (*p* < 0.05). However, no significant changes were observed in P-AKT protein level in HepG2-LEM construct (Figure 5E,F).

#### 3.2.4. ^188^ReO_4_-Treated Cells Did Not Initiate or Develop Tumor in Nude Mice

A total number of 5 × 10^6 188^ReO_4_-treated Huh7 cells and non-treated Huh7 cells were percutaneously injected to the flank of nude mice. Treated cells were already exposed to 37 MBq for 24 h. After 14 days, recipient animals were sacrificed (Supplementary Figure S1B). The nude mice received treated Huh7 cells did not have any tumor in the site of injection. However, the non-treated cells developed visible and palpable mass in the right flank of the recipient nude mice.

## 4. Discussion

Rhenium-188 is produced by a ^188^W/^188^Re generator conveniently and inexpensively in hospitals [23]. Different derivatives of ^188^ReO_4_, which are transarterialy injected to the HCC tumors, are under development as a targeting radionuclide for therapeutic purposes [24].

In past decades, cancer cell lines have played essential roles in revealing molecular mechanisms used in metabolism of new candidates in drug discovery [25]. The rationale for using cancer cell lines as an experimental model is that cancer cell lines retain the hallmarks of the primary cancer cells [26]. Although two-dimensional (2D) cell culture systems are efficient and reliable, they lack appropriate cell-cell and cell-ECM contact compared to the in vivo models. It has been demonstrated that 3D organotypic human cancer cell models are suitable alternatives to mimic the tumor microenvironment [27,28].

The present study assessed the therapeutic effect of ^188^ReO_4_ on hepatic cancer cell lines in 2D and 3D culture systems and in a mouse model. Data showed that dose and time of exposure to ^188^ReO_4_ affected the viability rate of cancerous cells. In addition, IC50 doses and dead cell percentages were different between 2D and 3D cultured cells. The IC50 value for Huh7 cells was 37 MBq, 18, 24, and 48 h after exposure to ^188^ReO_4_, whereas, in HepG2 cells, it was 55 MBq, 48 h post-exposure. These findings aligned with the flow cytometric results for the cell cycle phases in both cell lines. Flow cytometric analysis showed a significant dose and time-dependent reduction in the number of HepG2 cells in the S phase, while the number of Huh7 cells in the G2/M phase increased. Other studies demonstrated that rhenium components-induced cell cycle arrest at the G2/M phase, and this was mediated by inhibiting the phosphorylation of Aurora-A kinase [29,30]. In our study, cell cycle analysis showed a dose-dependent block in the G2/M phase that was in line with the results of Gilbertz and colleagues [31]. In addition, the percentages of emerged colonies in 2D culture were decreased significantly in both Huh7 and HepG2 cells after treatment with effective doses of ^188^ReO_4_.

The viability percentage was higher than 60% for HDF used as normal cells. Live and dead staining of Huh7 and HepG2 cells indicated that ^188^ReO_4_ treatment was effective on the mortality rate of both cells, whereas HDF cells did not have the same percentage of cell death [22]. Information on the toxicity of radionuclides on the normal tissues is more limited than that of external beam radiation and appears to be more variable [32]. Interestingly, we showed that the effective dose of ^188^ReO_4_ for HCC cell lines was well-tolerated in fibroblasts as normal cells [33]. The tolerance of normal tissues to radiation is higher compared to the cancerous cells but more variable. However, this variation is mainly attributed to the differences in the dosimetry method and the heterogeneous distribution of the radionuclides [32]. Heterogeneity of dose absorption at the cellular level remains a concern for therapies based on radionuclides [34]. The Wellcome Trust Case Control Consortium, at www.wtccc.org.uk (accessed on 18 November 2021), continues to study genetic differences in a large population of healthy and patients. Their data could enable researchers to perform meta-analysis for various models to reach the best estimate of tolerance in normal and cancer cells [32].

Limitations of 2D culture system include the lack of tissue-specific architecture and the altered or absent cell–cell and cell–ECM interactions that could contribute to the development or maintenance of tumors [35]. In 3D culture condition, the effective dose calculated for Huh7-LEM and HepG2-LEM was 55 MBq 18, 24, and 48 h after exposure. IC50 for Huh7-LEM was higher than 2D culture, while the effective dose for HepG2 cells was the same in 2D and 3D. Previous studies showed that tumor cells located inside the scaffolds were exposed to lower doses of therapeutic agents since the diffusion of therapeutic agents into the tumor mass (core) is limited [36]. Various studies demonstrated that enrichment of microenvironment with ECM components enhanced the viability of normal and tumor cells after treatment with radionuclides [37]. The mean percentage of proliferative cells in 3D treated groups compared to the control groups was 6.5 and 7.9% for Huh7 and HepG2, respectively. In HCC-LEMs, the decreased in proliferation rate indicated that ^188^ReO_4_ penetrated well in the 3D structures and affected cancer cells. 

Overall, cell proliferation was affected by the effective radiation dose and exposure time. The HUh7 and HepG2 cells treated with less than 37 or 55 MBq, as their IC50 dose, did not show any significant growth inhibition after 48 h.

Evidence to proof apoptosis induction after ^188^ReO_4_ exposure was obtained from the results of the flow cytometric, qRT-PCR, and western blot experiments. The Annexin/PI flow cytometric results showed that DNA damage induced by 55 MBq of ^188^ReO_4_ significantly increased apoptosis 48 h post-exposure in HepG2 cells.

Radiotherapy is one of the most effective modalities for cancer treatment, and P53 is a key molecule involved in the cellular response to ionizing radiation [38,39]. Given the importance of P53 expression in developing a response to the treatment in many types of tumors and determining the effectiveness of radiotherapy in different HCC cell lines, we evaluated P53 expression [40,41]. However, *p53* mRNA expression differs significantly between the two lines. ^188^ReO_4_ induced *p53* mRNA expression in Huh7 cells after 24 h but not in HepG2 cells. Immunoblotting results showed increased P53 protein expression in Huh7 cells after exposure to 37 MBq of ^188^ReO_4_ but no significant changes in this regard in HepG2 cells after exposure to 55 MBq. The apoptosis activated by induction of Bax, P53, and caspase-mediated cleavage of p21 is an important radiation-induced cell death mechanism in cancer cells [42]. To confirm if P53 protein triggered apoptosis after irradiation in both cell lines, *BAX* mRNA and protein expressions were evaluated. It was showed that *BAX* mRNA up-regulated 24 and 48 h post-exposure in Huh7 cells, and 24 h after exposure in HepG2 cells. Bax protein expression induction was significant in Huh7 cells, while its protein expression remained unchanged in HepG2 cells. Since protein expression of BAX in HepG2 cells was not changed, apoptosis might be due to the direct effect of O_2_ free radicals [43]. Reactive oxygen species like HO_2_^}^ or OH^}^ can cause Ca^2+^ release from the mitochondria, provoking various pro-apoptotic consequences [44,45].

The qRT-PCR data showed significant differences in *p53* mRNA expression in Huh7-LEM treated with 55 MBq after 48 h compared to the control group, while, in HepG2-LEM, no notable changes in *p53* mRNA expression were noticed. The IF analysis of p53 showed apoptosis induction after ^188^ReO_4_ treatment in Huh7-LEM but not in HepG2-LEM. *Bax* mRNA expressions in ^188^ReO_4_-treated HCC-LEM were upregulated 24 and 48 h post-treatment in Huh7-LEM and 48 h in HepG2-LEM. These findings demonstrated that ^188^ReO_4_ could induce apoptosis and inhibit proliferation in HCC cells, providing convincing evidence for its clinical application. 

The mRNA and protein expression of p53 was not changed in cells cultured on 3D LEM compared to the 2D cultured cells. This trend was also observed for *Bax* mRNA and protein expression in the 2D culture of Huh7 and Huh7-LEM. *p53* mRNA and protein expression in 2D and 3D had the same pattern, whereas the opposite results for Bax mRNA and protein expression were obtained in 2D compared to 3D. In the case of *Bax* mRNA and protein expression in 2D and 3D, the microenvironment conditions in LEM 3D culture could induce significant changes in cellular behavior compared to conventional 2D culture, which is consistent with previous studies on microenvironment-mediated factors [46,47]. This study showed that ^188^ReO_4_ induced apoptosis and cell cycle arrest in HCC cells in a dose- and time-dependent manner in both 2D and 3D cultures.

The PI3K/AKT signaling pathway has been extensively studied and demonstrated to be essential for radiotherapy resistance in various cancers [48,49]. DNA-dependent protein kinase (DNA-PK) is a protein complex essential for the double-strand break (DSBs) repair [50]. Phosphorylation of DNA-PKcs (T2609) is related to DSB repair and radiosensitivity of cells [50]. It has been reported that the PI3K/Akt pathway is involved in regulating DNA-PKcs [51]. In the present study, it was observed that mRNA expression of *PTEN* as a PI3K/AKT inhibitor and PI3K was not changed after exposure to effective dose of ^188^ReO_4_ in 2D culture systems. Western blot data showed that AKT-P protein significantly decreased in both Huh7 and HepG2 cells after exposure.

We did not observe any considerable changes in *PTEN* mRNA expression in both Huh7- and HepG2-LEM, 3D constructs, whereas *PI3K* mRNA expression increased 24 and 48 h post-exposure in Huh7-LEM and 18, 24, and 48 h after treatment in HepG2-LEM. Immunoblotting results showed a significant increase in AKT-P protein expression in treated Huh7-LEM but remained unchanged in HepG2-LEM. Previous studies suggested that the scaffold can increase resistance to radionuclide treatment in HCC 3D culture systems [52]. Several reports indicated that decellularized scaffolds are useful in drug discovery [53]. The natural ECM enables HCC cells to sustain their original capabilities in terms of drug metabolism and proliferation [53]. Therefore, HCC cells cultured in decellularized 3D scaffolds can provide a better platform for drug toxicity assessments than conventional 2D culture systems [53,54].

## 5. Conclusions

In this study, we developed 2D, 3D, and in vivo model of HCC to evaluate the therapeutic effects of ^188^ReO_4_. Although IC50s of ^188^ReO_4_ vary in different models of each cancerous cell line, our study indicated that treatment with ^188^ReO_4_ had remarkable impacts on proliferation inhibition. It seems that different responses to irradiation depends on initial differences in the cell’s heterogenecity and genetic discrepancy, microinvironment variations, and in vivo condition in different models. Generally, cell viability was time- and dose-dependent in all models. Using immunoblotting and immunofleurscence analysis, we have shown that ^188^ReO_4_ radiation induced apoptosis by alterations in the levels of bax, P53, and Caspase 3. We demonstrated that ^188^ReO_4_ (37 MBq) exposure resulted in significant cell cycle arrest in Huh7 cells at the early time-point of 24 h. Similarly, growth suppression and prolifration inhibition were observed following exposure to ^188^ReO_4_, providing adequate evidence for its potential clinical use for HCC radiotherapy.

This study also confirmed, the role of PI3K-Akt signaling pathway in resistance to irradiation. Interestingly, we observed differences in p-AKT protein expression after treatment. We suggest that the ECM, as the cells scaffold, can have a remarkable role in radio-resistance. In adition, we revealed that treatment with ^188^ReO_4_ induced a significant anti-tumor activity in-vivo and showed limited and declined tumor formation capacity of HCC cells in nude mice. We herein used two different cell lines of Huh-7 and HepG2 as a preliminaty platform to assess the effects of ^188^ReO_4_ administration on HCC cells and consider the individual differences in response to treatment. Further research using an array of cell lines is required to confirm the findings and find reproducibility between cell lines.

Furthermore, this study provided a preliminary roadmap for finding comprehensive data for studying radiobiology of therapeutic radionuclides in HCC treatment. However, more studies with further cell lines and animal models are needed to explore the impact of ^188^ReO_4_ on HCC treatment from both molecular and cellular point of view. Moreover, assessing the effects of ^188^ReO_4_ in combination with immunotherapeutic approaches seems necessary for more selective and personalized therapy.

## Data Availability

The data supporting the findings are available.

## Supplementary Materials

The following supporting information can be downloaded at: https://www.mdpi.com/article/10.3390/cells11020305/s1, Figure S1. Tumor formation assay in the nude mice model. (**A**) Huh7 cells were inoculated into nude mice (*n* = 1) at 3 × 10^6^, 5 × 10^6^, 7 × 10^6^, 10 × 10^6^ cells subcutaneously. Representative tumors for the mentioned cells were shown. The size of tumors was in correlation with the number of initial injected cells. (**B**) Tumor established in the control nude mice after injection of 5 × 10^6^ Huh7 cells (left). Tumors were not established using treated cells in nude mice (right, *n* = 3).

## Institutional Board Review Statement

The animal experiments were approved by the Local Ethical Committee of the Royan institute (approval No. IR.ACECR.ROYAN.REC.1397.052).
