# Correction: Asadian et al. Rhenium Perrhenate (^188^ReO4) Induced Apoptosis and Reduced Cancerous Phenotype in Liver Cancer Cells. *Cells* 2022, *11*, 305

**DOI:** 10.3390/cells13171456

**Published:** 2024-08-30

**Authors:** Samieh Asadian, Abbas Piryaei, Nematollah Gheibi, Bagher Aziz Kalantari, Mohamad Reza Davarpanah, Mehdi Azad, Valentina Kapustina, Mehdi Alikhani, Sahar Moghbeli Nejad, Hani Keshavarz Alikhani, Morteza Mohamadi, Anastasia Shpichka, Peter Timashev, Moustapha Hassan, Massoud Vosough

**Affiliations:** 1 Cellular and Molecular Research Center, Research Institute for Prevention of Non-Communicable Diseases, Qazvin University of Medical Sciences, Qazvin 34199153, Iran; samieh.asadian@gmail.com (S.A.); haematologicca@gmail.com (M.A.); smoghbelinrjad@qums.ac.ir (S.M.N.); 2 Department of Regenerative Medicine, Cell Science Research Center, Royan Institute for Stem Cell Biology and Technology, ACECR, Tehran 16635148, Iran; alikhanim81@gmail.com (M.A.); hani.keshavarz865@gmail.com (H.K.A.); 3 Department of Biology and Anatomical Sciences, School of Medicine, Shahid Beheshti University of Medical Sciences, Tehran 16123798, Iran; piryae_a@yahoo.com; 4 Department of Tissue Engineering and Applied Cell Sciences, School of Advanced Technologies in Medicine, Shahid Beheshti University of Medical Sciences, Tehran 16123798, Iran; 5 Department of Organic Chemistry, Karaj Branch, Islamic Azad University, Karaj 16255879, Iran; b_akalantari@yahoo.com; 6 Faculty of Nuclear Engineering, Shahid Beheshti University, Tehran 15689456, Iran; behzad_davar2001@yahoo.com; 7 Department of Internal Medicine N1, Sechenov University, 119991 Moscow, Russia; kapustina_v_a@staff.sechenov.ru; 8 Department of Physical Chemistry, Faculty of Science, University of Tehran, Tehran 17456987, Iran; sarvar20102010@gmail.com; 9 World-Class Research Center “Digital Biodesign and Personalized Healthcare”, Sechenov University, 119991 Moscow, Russia; ana-shpichka@yandex.ru; 10 Institute for Regenerative Medicine, Sechenov University, 119991 Moscow, Russia; 11 Chemistry Department, Lomonosov Moscow State University, 119991 Moscow, Russia; 12 Experimental Cancer Medicine, Institution for Laboratory Medicine, Karolinska Institute, 141-83 Stockholm, Sweden; Moustapha.Hassan@ki.se; 13 Clinical Research Center, Karolinska University Hospital Huddinge, 141-83 Stockholm, Sweden

In the original publication [1], there was a mistake in Figure 1A as published. The two upper-right images were repeated unintentionally in the neighboring boxes. In addition, the control figures on the left side overlapped. Figure 1A has been corrected and appears below. The authors state that the scientific conclusions are unaffected. This correction was approved by the Academic Editor. The original publication has also been updated.

## Figures and Tables

**Figure 1:**
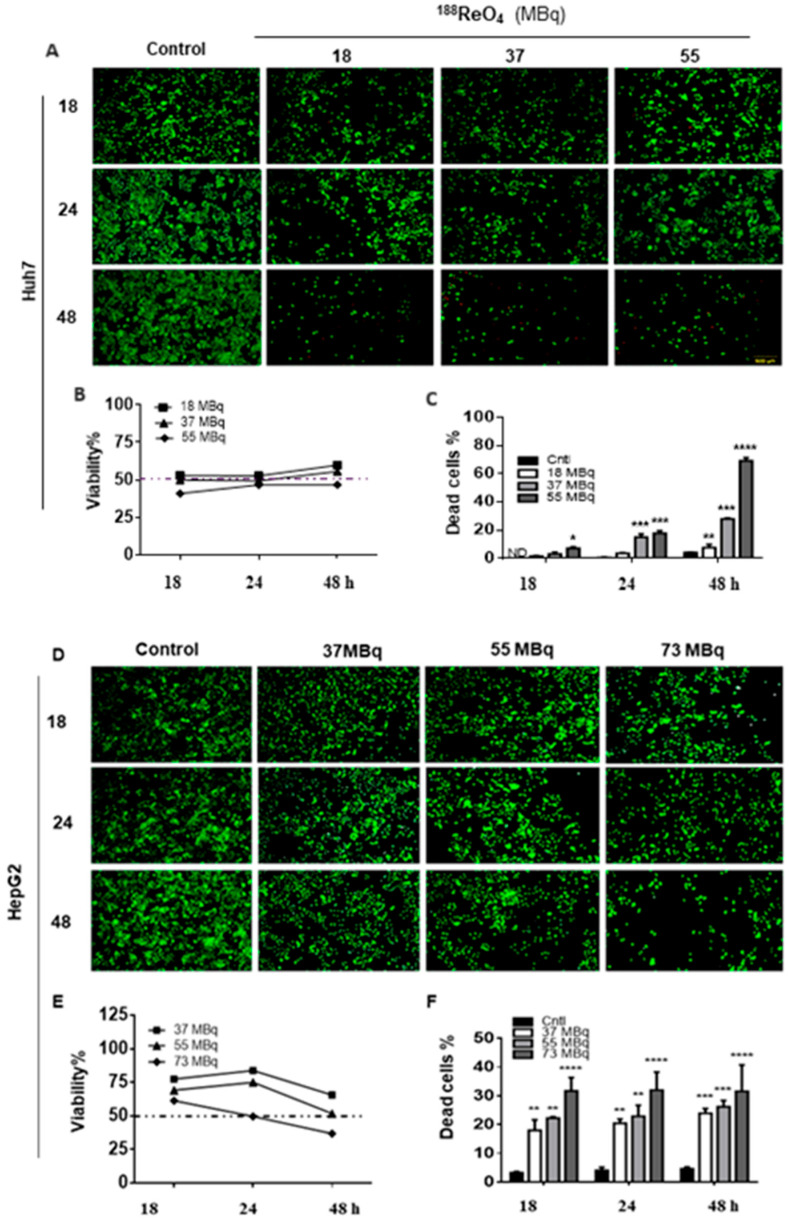
^188^ReO_4_ IC50 dose finding on Huh7 and HepG2 cell lines. Huh7 cells viability was measured using LIVE/DEAD^®^ Viability/Cytotoxicity Kit and the mean viability of untreated cells (control group), and the treated groups were compared on various doses of 18, 37, and 55 MBq of ^188^ReO_4_ at 18, 24, and 48 h post-exposure for finding the effective dose of ^188^ReO_4_ (**A**–**C**). HepG2 cells viability was measured using LIVE/DEAD^®^ Viability/Cytotoxicity Kit in response to 37, 55, and 73 MBq of ^188^ReO_4_ 18, 24, and 48 h post-exposure for finding the effective dose of ^188^ReO4 in treated HepG2 cells (**D**–**F**). The IC50 value of 188ReO_4_ in Huh7 cells was 37 MBq 24 h after exposure, and it was 55 MBq 48 h for HepG2 cells. Data are presented as the mean ± SD, *n* = 3 (* *p* < 0.05, ** *p* < 0.01, and *** *p* < 0.001, **** *p* < 0.0001).
