# Supplementary material for: Rhenium Perrhenate (188ReO4) Induced Apoptosis and Reduced Cancerous Phenotype in Liver Cancer Cells
Source: Cells. 2022 Jan 17;11(2):305. doi: 10.3390/cells11020305 (PMC8774126; doi:10.3390/cells11020305)
Supplement: Supplementary file 1 [file cells-11-00305-s001.zip › cells-1494780-supplementary.pdf]

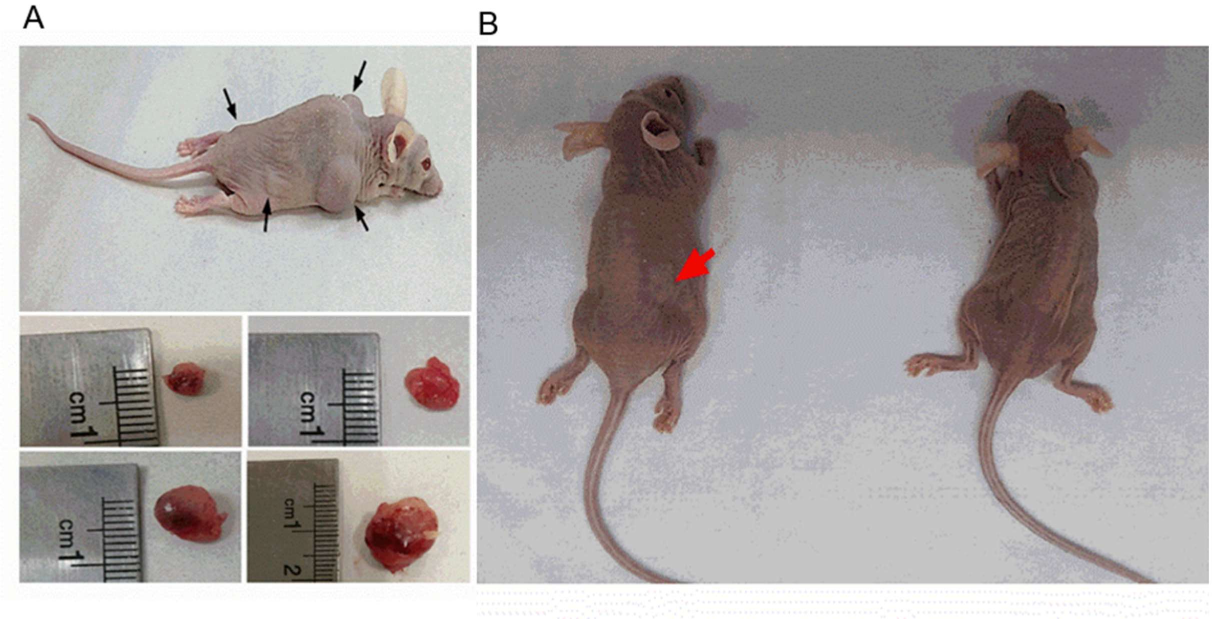


**Supplementary Figure S1.** Tumor formation assay in the nude mice model. (**A**) Huh7 cells were inoculated into nude mice ($n$ = 1) at 3 × 106, 5 × 106, 7 × 106, 10 × 106 cells subcutaneously. Representative tumors for the mentioned cells were shown. The size of tumors was in correlation with the number of initial injected cells. (**B**) Tumor established in the control nude mice after injection of 5 × 106 Huh7 cells (left). Tumors were not established using treated cells in nude mice (right, $n$ = 3).
